# Taste Masking Study Based on an Electronic Tongue: the Formulation Design of 3D Printed Levetiracetam Instant-Dissolving Tablets

**DOI:** 10.1007/s11095-021-03041-9

**Published:** 2021-05-11

**Authors:** Zengming Wang, Jingru Li, Xiaoxuan Hong, Xiaolu Han, Boshi Liu, Xianfu Li, Hui Zhang, Jing Gao, Nan Liu, Xiang Gao, Aiping Zheng

**Affiliations:** 1 State Key Laboratory of Toxicology and Medical Countermeasures, Beijing Institute of Pharmacology and Toxicology, 27th Taiping Road, Haidian District, Beijing, 100850 China; 2 School of Pharmacy, Xuzhou Medical University, Xuzhou, 221000 China

**Keywords:** Binder jet 3D printing, electronic tongue, instant-dissolving tablets, principal component analysis, taste-sensing system

## Abstract

**Purpose:**

Proper taste-masking formulation design is a critical issue for instant-dissolving tablets (IDTs). The purpose of this study is to use the electronic tongue to design the additives of the 3D printed IDTs to improve palatability.

**Methods:**

A binder jet 3D printer was used to prepare IDTs of levetiracetam. A texture analyzer and dissolution apparatus were used to predict the oral dispersion time and *in vitro* drug release of IDTs, respectively. The palatability of different formulations was investigated using the ASTREE electronic tongue in combination with the design of experiment and a model for masking bitter taste. Human gustatory sensation tests were conducted to further evaluate the credibility of the results.

**Results:**

The 3D printed tablets exhibited rapid dispersion (<30 s) and drug release (2.5 min > 90%). The electronic tongue had an excellent ability of taste discrimination, and levetiracetam had a good linear sensing performance based on a partial least square regression analysis. The principal component analysis was used to analyze the signal intensities of different formulations and showed that 2% sucralose and 0.5% spearmint flavoring masked the bitterness well and resembled the taste of corresponding placebo. The results of human gustatory sensation test were consistent with the trend of the electronic tongue evaluation.

**Conclusions:**

Owing to its objectivity and reproducibility, this technique is suitable for the design and evaluation of palatability in 3D printed IDT development.

## Introduction

3D printing is a promising technology used for the fabrication of personalized pharmaceutical dosage forms. It is based on digital models that help construct objects via layer-by-layer printing, and finally turns the digital blueprints into physical objects. Binder jet 3D printing (BJ-3DP), also known as drop-on-powder 3D printing, is more widely used in the pharmaceutical industry compared to other 3D printing technologies [18]. The first and only 3D printed drug using BJ-3DP – Spritam®, a landmark in 3D printing technology of pharmaceutical research, was approved by FDA in 2015 [14]. Printing ink containing liquid binder is loaded into the printing head and jetted on a powder bed in precise path and dose, and the printing processes will keep repeating to produce the desired 3D product [5, 22]. Through the layer-by-layer bonding of printing ink and powder, the technology allows for the preparation of instant-dissolving tablets (IDTs) with a highly porous internal structure that can be rapidly dispersed in water, thus solving the problem of dysphagia in patients.

One of the most important issues with oral IDTs is their palatability in the oral cavity. 3D printed IDTs can be dissolved in water or taken directly with small sip of water. Regardless of the method of oral administration, the drug is released quickly and in full contact with the tongue, resulting in a more bitter taste compared to traditional tablets. Taste of formulations is crucial for patient compliance, especially with pediatric formulations, where taste is one of the primary determinants of market performance and commercial success of oral pharmaceuticals. Therefore, IDTs must be scrutinized more closely for palatability, and unpleasant taste should be detected and masked in final preparations, for example, by adding sweeteners and/or flavoring agents [17].

Taste is an important sensory characteristic that determines the acceptability of oral products. Biologically, taste transduction is mediated by specialized neuroepithelial cells, referred to as the taste receptor cells, that are organized into groups of 40–100 and form taste buds. Different taste modalities function by different transduction mechanisms [23, 24]. Salty taste is mediated by sodium ion flux through the apical sodium channels [2], while sour taste is mediated via the blockade of hydrogen ions by potassium or sodium channels [7]. Sweet and bitter tastes are transduced via G protein-coupled receptors [6]. Nevertheless, taste transduction mechanisms are complex and not fully elucidated [24].

Oral tasting is the most commonly used method to evaluate taste; however, the results depict poor reproducibility owing to individual variability and personnel subjectivity. The method also has limitations owing to the possible toxic side effects of drugs [1]. Taste assessment using laboratory animals, especially mammals, has been suggested as an alternative to human tasting. This approach has certain limitations related to the availability of animals with flavor perception similar to humans, as well as ethical considerations [17]. Currently, rodents such as mice and rats are generally being used for taste evaluation studies. Notably, although laboratory animals can be used to evaluate flavor acceptability, their inability to describe sensory characteristics limits appropriate differentiation between formulations. Owing to these problems, a taste-sensing system (such as an electronic tongue) can be a safe and objective alternative.

The electronic tongue is an intelligent instrument used for taste analysis, which is based on bionics, and is capable of evaluating the masking effect through specific sensor membranes and electrochemical techniques. It has already been utilized in taste evaluation and design of pharmaceutical formulations, which may reduce the bias in the results obtained through *in vivo* evaluations caused by subjective differences and ethical issues [9, 12, 15, 16]. The concept of the electronic tongue can be described like the human being. The working principle is based on biological recognition, in which information is gathered using arrays of non-specific sensors in the nose or tongue, and the data is subsequently processed by the brain. The electronic tongue mimics these processes using chemometric methods and artificial intelligence, i.e., it can discriminate, identify, and/or quantify a sample [3, 13, 20]. From an analytical point of view, it comprises different sensors with varying properties and characteristics of partial selectivity or cross-selectivity; the ability of these sensors to measure and characterize complex liquid matrices makes them unique in the field of analytical systems [23]. These sensors transduce the potential of the membrane into an electronic signal and the trapping of ions or molecules on the chemically sensitive layer generates a change in the membrane potential. This change leads to finally a variation of potential between the source and drain region of the field effect transistor(potentiometric measurement) of the sensor.

The sensor array has been designed to provide relative information of the following taste attributes (when relevant): sourness, saltiness and umami, directly based on a defined specific sensor. In addition, there are several general-purpose cross-sensing sensors. Used with a defined methodology based on standard addition methodology, this specific sensor array allows providing relative information of the other taste attributes (when relevant), such as astringency, metallic, spicy and so on. The relationship between these tastes and a sensor is done in-situ based on the corresponding analysis. This methodology allows to rank samples according to the tastes of interest and define taste according to the products analyzed. In both cases, the final result obtained is a relative unit score of taste. On a given taste attribute axis, the relative positioning of the different samples allows ranking them according to this taste perception.

In this study, levetiracetam was chosen as the model drug, and BJ-3DP was used to prepare the 3D printed IDTs with a loose internal structure and extremely high porosity. A texture analyzer and dissolution apparatus were used to predict the dispersion time in the oral cavity and *in vitro* drug release of the tablets, respectively. As a first-line antiepileptic drug, levetiracetam is used in large doses and has a strong bitter taste; therefore, an appropriate design for a taste-masking formulation is essential. The palatability of different formulations with sucralose as a sweetener and spearmint as a flavoring agent was investigated using the ASTREE electronic tongue in combination with design of experiments (DoE) and a model for masking the bitter taste. This study demonstrates that the electronic tongue with its seven taste sensors can be utilized to design taste-masking formulations and evaluate 3D-printed IDTs to meet individual requirements.

## Materials and Methods

### Materials

Levetiracetam was purchased from Zhejiang Apeloa Jiayuan Pharmaceutical Co., Ltd. (China). Microcrystalline cellulose (MCC PH101) and mannitol (Pearlitol 50C) were purchased from Asahi Kasei Corporation (Japan) and Roquette Frères (France), respectively. Spearmint flavor and sucralose were provided by Kerry Group (Ireland) and Alpha Hi-Tech (China), respectively. Colloidal silicone dioxide (Aerosil 200) and polyvinylpyrrolidone (PVP K-30) were provided by Evonik Degussa GmbH (Germany) and BASF (Germany), respectively. Glycerin was purchased from Nanchang Baiyun Pharmaceutical Co., Ltd. (China). All the solvents were of analytical grade.

### Preparation and Characterization of the 3D-Printed Tablets

#### Powder Mixture and Printing Ink

Levetiracetam, being the active pharmaceutical ingredient (API), accounted for 65% of the powder. MCC PH101 and Pearlitol 50C were used as fillers. Spearmint flavor was used as a flavoring agent and sucralose was used as a sweetener. Aerosil 200 was added to improve the fluidity of the powder. Blending of the above components was performed using a Hopper Mixer (HSD15 Lab Mixer, Canaan Technology, China) at 20 rpm for 20 min to obtain the final powder mixture. The quantity of the additives were adjusted as required. The printing ink consisted of 40% (*v*/v) isopropanol aqueous solution containing 0.05% (*w*/w) PVP and 4% (w/w) glycerin.

#### Design of the Dosage Form and Printing Process

**Fig. 1:**
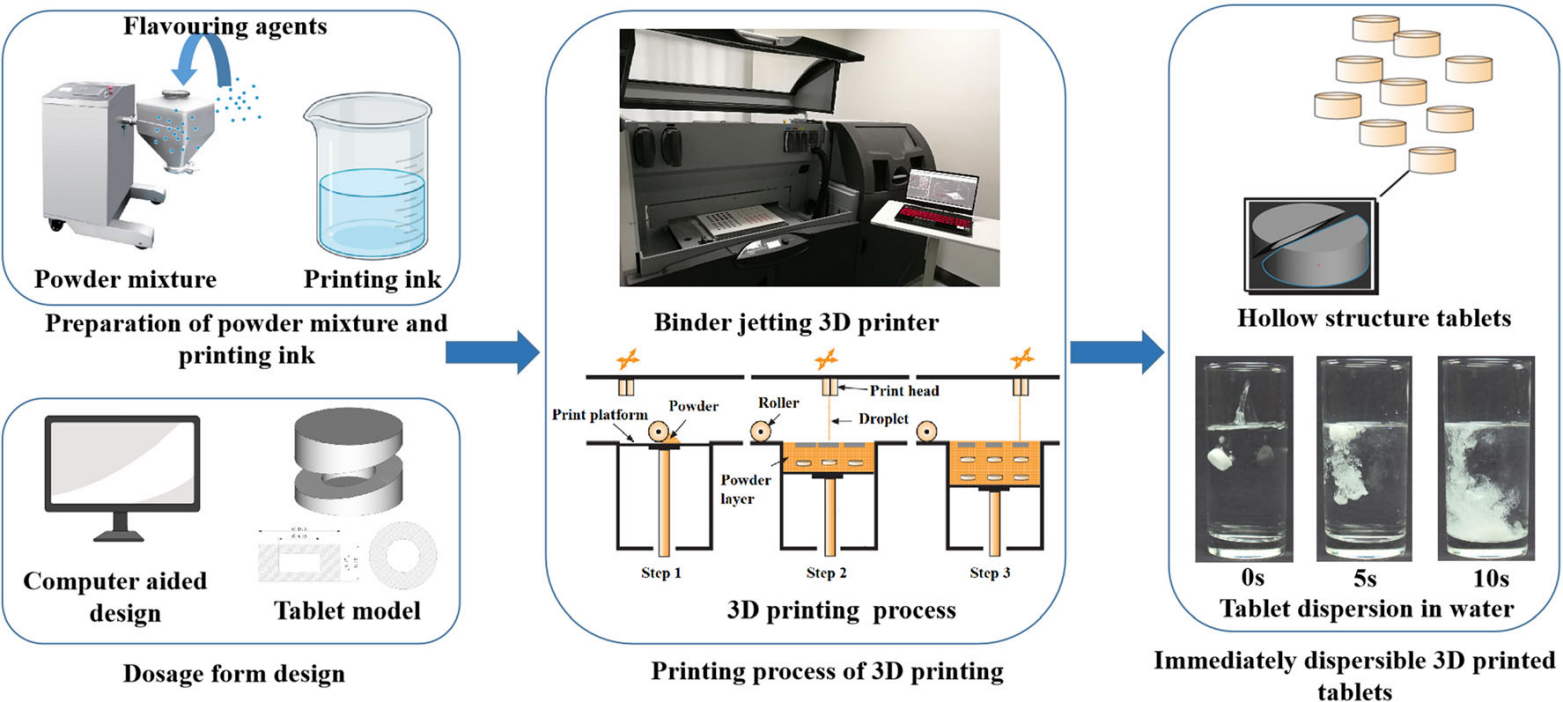
Schematic diagram of the printing process of 3D-printed instant-dissolving tablets using a binder jet 3D printer.

3D Sprint (3D systems, USA), a computer-aided design software, was used to create 14.6 mm (diameter) × 6.57 mm (height) round model of tablets (Fig. 1). The designed tablet weighed 770 mg, and had a strength of 500 mg. Based on the flexibility and accuracy of the 3D printing technology, the tablet size was adjusted to achieve a specific strength, such as 1000 mg (18.5 mm × 8.33 mm), 750 mg (16.8 mm × 7.56 mm), 250 mg (11.5 mm × 5.18 mm).

As shown in Fig. 1, printing was conducted using a BJ-3DP (ProJet CJP 660 Pro, 3D systems, USA). The file of the designed model was uploaded into the software of the 3D printer that sliced the model and sent the slices to the 3D printer. Thin layers of the powder mixture (100 μm per layer) were spread across the platform. The print carriage moved across each layer; using a hot-bubble printing head, ink droplets were selectively ejected onto a specific area, as directed. The printing head had 304 nozzles, and a single droplet from the nozzle was 18 pL. The ink solidified the powder only in the cross-section of the designed model, and the remaining powder was used for support. After printing, the tablets were dried at 40°C for 3 h to remove organic solvents and excess moisture, and the support powder was recycled through an integrated vacuum system. The tablets were then cleaned with an air brush to remove excess powder.

#### Prediction of Dispersion Time in the Oral Cavity and *In Vitro* Drug Release

Generally, the 3D printed IDTs were placed in the oral cavity (on the tongue) with a sip of water (approximately 15 ml) to take the medicine. A texture analyzer (TA touch, BosinTech, China) was used to predict the dispersion time of the tablets in the oral cavity. In the experiment, a constant pressure was employed, and the dispersion curve of the tablets of different strengths in 3 ml purified water was measured within 50 s. The parameters were as follows: pretest speed 5 mm/s; test speed 8 mm/s; trigger force 5 gf; and target pressure 50 g (1 gf ≈ 0.0098 N). *In vitro* drug release was determined using a USP II dissolution apparatus (RC806D, Tianfa Technology Co., Ltd., China) in 900 ml phosphate buffer solution (pH 6.8) at 37°C with a paddle speed of 50 rpm. The drug release of 6 tablets of each strength was measured after 2.5, 5, 10, 15, 20, and 30 min and analyzed using high-performance liquid chromatography (HPLC).

### 3^2^ Full Factorial Design of Experiments (DoE)

In this study, 3D printed IDTs with good palatability was achieved by adjusting the quantity of sucralose and spearmint flavor using an electronic tongue. A two-factor, three-level full factorial design, as shown in Table I, was used to study the effects and interactions of both factors on the response of the electronic tongue. The maximum quantity of sucralose and spearmint flavor was limited to 2% and 0.5%, respectively, based on the maximum weight of the IDTs and FDA regulations on inactive ingredients (IIG) [4]. In addition, the API (sample no. 10) and the corresponding placebos were measured as reference samples. In the plot, the relative distances of the samples for the designed experiment from the API and the corresponding placebo were used as response indicators based on the results of a principal component analysis (PCA) [11, 19] of the signals from the electronic tongue.

**Table I:** 3^2^ Full Factorial Design to Study the Quantity of the Additives in the 3D Printed Tablets Using an Electronic Tongue

Factors: Quantity of the additives in the 3D-printed tablets		Levels		
		1	2	3
X1	Sucralose (%)	0.5	1	2
X2	Spearmint flavor (%)	0.2	0.3	0.5
Responses of the electronic tongue		Goal		
Y1	Relative distance from the API	Maximize		
Y2	Relative distance from the placebo	Minimize		

### Taste Evaluation by Electronic Tongue

#### Equipment and Principle of Measurement

**Fig. 2:**
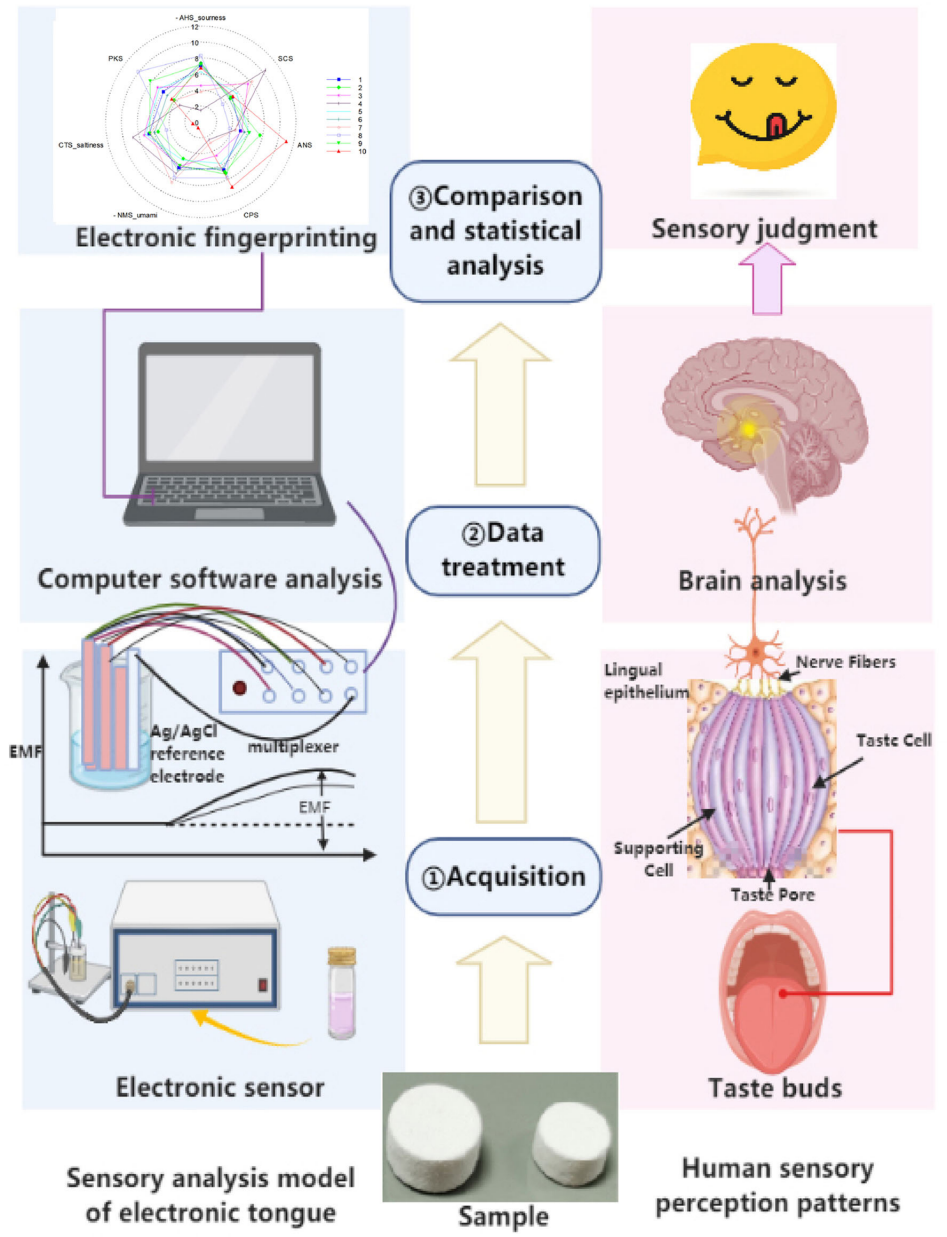
Comparison of the human sensory system and the principle of taste evaluation by the electronic tongue.

An electronic tongue (ASTREE, Alpha MOS, France) was used to screen the quantity of the additives to achieve the best taste. ASTREE is a detection instrument based on bionics that is used to analyze and identify the taste of liquids. It is primarily composed of three parts: a sampling system, sensor system, and data processing system. It can meet the requirement of the objective analysis of the quality of taste of the liquid and distinguish the overall difference in taste between the samples. ASTREE has seven types of cross-inductance sensors, including AHS, PKS, CTS, NMS, CPS, ANS, and SCS, which is equivalent to a seven-dimensional space. AHS, CTS, and NMS are sourness, saltiness, and umami sensors respectively, while PKS, CPS, ANS, and SCS are general-purpose sensors. These sensors are modified solid electrochemical electrodes based on well-established technology: Chemical Sensitive Field Effect Transistor (ChemFETs). As other solid sensors, potentiometric sensors consist in two essential parts: a transducer and a chemically sensitive layer. Ag/AgCl was selected as the reference electrode. The principle of taste evaluation of the electronic tongue is shown in Fig. 2. The organic membrane on the sensor is highly sensitive to ionic and neutral compounds, and the response signal is obtained by measuring the difference in electronic potential between the sensor and the reference electrode. The trapping of ions or molecules on the chemically sensitive layer generates a change in the membrane potential. This change leads to finally a variation of potential between the source and drain region of the field effect transistor (potentiometric measurement) of the sensor. Following the analysis of the signal value, we compared the relative intensities of both the additives in the samples on a scale of 0–12 to determine whether the samples can be distinguished well, and to rank the intensities of the additives.

Due to the complexity of the sweet and bitter substances, we conducted a PCA of the results obtained from each sensor, for a comprehensive judgment. PCA was used to perform a linear transformation and reduce the dimensionality to a two-dimensional space for easy observation and analysis. The transverse axis was principal component 1 (PC1), and the longitudinal axis was PC2 (PC1 + PC2 + … + PC7 = 100%; PC1 and PC2 represent the comprehensive information on the samples). When PC1 + PC2 > 80%, it is proved to be representative of most of the information on the samples.

#### Investigation of Levetiracetam Concentration in the Samples

The concentration of levetiracetam in the samples was determined to ensure whether this technique can distinguish the differences in taste between the samples well, and identify the levetiracetam concentrations to be tested in the designed experiment. The following three samples were prepared with different concentrations: (1) sample no. 10: 3 g of levetiracetam dissolved in 90 ml of water, which is equivalent to a tablet (500 mg) dispersed in 15 ml of water (33.3 mg/ml), (2) sample no. 10–1: 1.5 g of levetiracetam dissolved in 90 ml of water, which is equivalent to a tablet (500 mg) dispersed in 30 ml of water (16.7 mg/ml), and (3) sample no. 10–2: 0.9 g of levetiracetam dissolved in 90 ml of water, which is equivalent to a tablet (500 mg) dispersed in 50 ml of water (10 mg/ml).

The processed samples were placed in a beaker for anlaysis using the electronic tongue under the following conditions: sample volume 25 ml, collection time 120 s, and cleaning time 10 s. The outputs of the taste sensors were measured thrice for each sample. PCA and partial least square (PLS) regression analysis [8, 21] were used to discriminate the response signals and quantitatively analyze the concentration. PLS regression analysis is a multivariate statistical method for studying the relationships between variables; it helps calculate the regression coefficients of the variables according to their weightages and creates regression equations. A linear correlation between sensor response and drug concentration was obtained after PLS regression analysis, and predictions were made for the samples with an unknown intensity of bitterness.

#### Analysis and Evaluation of the Samples for the Designed Experiment

Sample no. 1–9 in Table II were prepared with a concentration of 33.3 mg/ml of levetiracetam and tested using the electronic tongue to determine the optimal quantity of the additives. The sample preparation method was as follows: 6 tablets (500 mg) were dissolved in 90 ml of distilled water; all the tablets completely dispersed within 30 s. After stirring, the supernatant was filtered, and the filtrate was placed in a beaker (25 ml) for testing using the electronic tongue. The experimental conditions were the same as those in mentioned in Section 2.4.2. The distance between the response signals of sample no. 1–9 and sample no. 10 were observed in the two-dimensional spatial image of the PCA; the farther the distance, the greater was the difference in taste.

**Table II:** Running Order for the Optimization of Quantity of the Additives

Running order (Sample no.)	Quantity of additives in the 3D-printed tablets	
	A:Sucralose (%)	B:Spearmint flavor (%)
1	0.5	0.5
2	0.5	0.2
3	2	0.2
4	2	0.5
5	1	0.2
6	0.5	0.3
7	2	0.3
8	1	0.5
9	1	0.3

Note: In the abovementioned samples, only the quantity of the additives varied, whereas the composition of the other ingredients remained the same

#### Model for Masking the Bitter Taste

A model for masking the bitter taste was developed to further determine the optimal quantity of the additives. The placebo samples (without API) were prepared at a concentration corresponding to the sample no. 1–9. Based on the response values obtained using the electronic tongue, PCA analysis was conducted to determine the response signal distances between sample no. 1–9 and the corresponding placebo samples. The closer the distance of the response signal, the better the bitterness masking effect. The experimental conditions were the same as those described in Section 2.4.2.

### Human Gustatory Sensation Test

This study was conducted in accordance with the ethical principles originating from the Declaration of Helsinki and was in compliance with local regulatory requirements. The test included 10 healthy adults (5 men and 5 women; mean age SD = 26.7 ± 2.3 years) who participated in the study after having signed informed consent. All participants were informed of all the details of the experiment. It was conducted in a randomized crossover, double-blind trial with the same sample preparation method as in “2.4.3”.

The visual analogue scale (VAS) is commonly used for assessing the levels of palatability or pain in human subjects, and several studies [10, 12] have shown that the VAS can be used as an evaluation criterion for oral tasting of preparations. Usually, a 10 cm vernier caliper with 10 scales is used, with 0 points indicating the best and 10 points representing the worst. The clinical assessment is ″0–2″ as “excellent”, ″3–5″ as “good”, ″6–8″ as “acceptable”, and “>8” as “poor”. In the test, all participants were asked to score the “overall palatability” using the 10 cm VAS by placing a mark after 3 ml of the sample has been tasted. The different samples were tested in 5-min intervals with adequate mouth rinsing in between.

## Results and Discussion

### Prediction of Dispersion Time in the Oral Cavity and *In Vitro* Drug Release

**Fig. 3:**
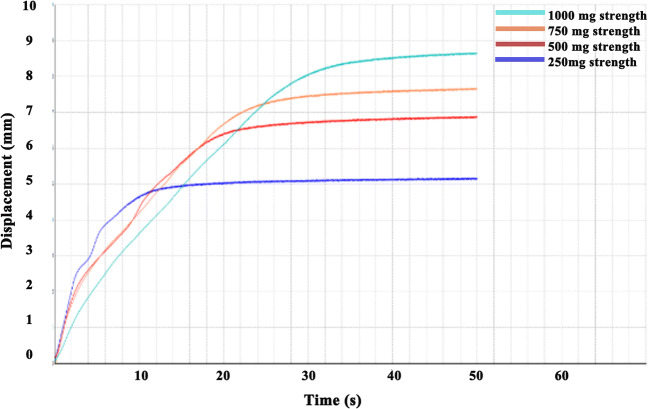
Dispersion curve of the 3D-printed tablets of different strengths determined using a texture analyzer at a constant pressure.

**Fig. 4:**
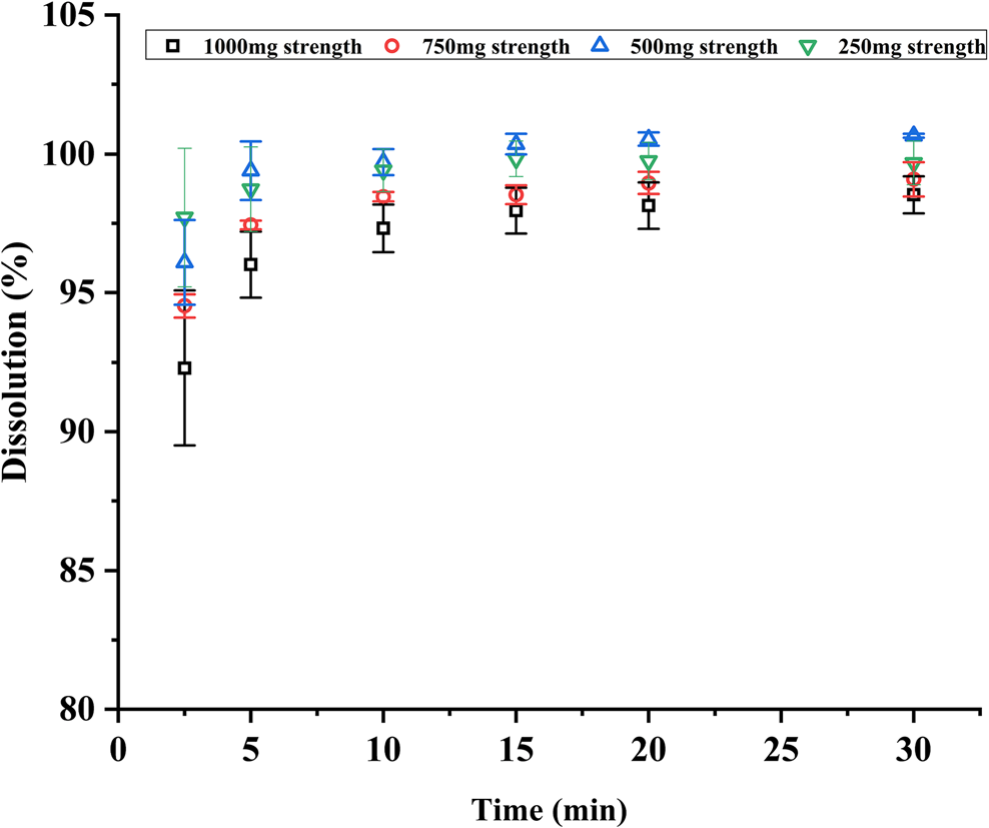
*In vitro* drug release profiles of the 3D-printed tablets of different strengths at pH 6.8 (*n* = 6).

In terms of the dispersion curve determined using the texture analyzer at a constant pressure shown in Fig. 3, the IDTs of different strengths showed a rapid dispersion time within 30 s. And the lesser the tablet strength, the shorter the dispersion time, which was related to the fact that smaller tablets can be wetted faster in water and dispersed quickly under slight force. The results of the *in vitro* drug release profiles in Fig. 4 show that the IDTs of different strengths exhibited a rapid release, and the drug was almost completely released after 2.5 min. These results demonstrated that the IDT prepared in this study could be rapidly dispersed in water, which can be attributed to its highly porous internal structure prepared using the BJ-3DP. Through a layer-by-layer printing process, BJ-3DP prepared tablets with a structure of loose interior and tight exterior, which could ensure strong mechanical properties and rapid dispersion characteristics simultaneously. This advantage of the IDTs is very convenient for patients with swallowing difficulties, such as children, to take medication, but it also places higher demands on taste-masking.

### Determination of Sample Concentration

#### Sensor Response and Taste Analysis

**Fig. 5:**
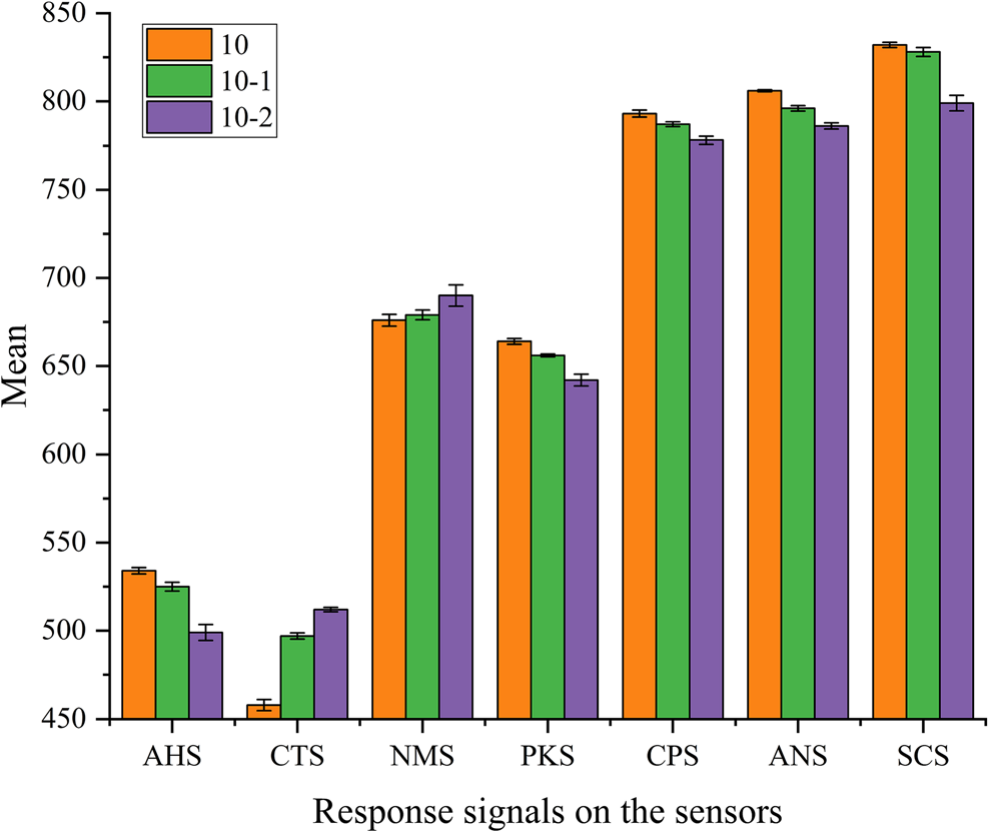
The response signals by different sample concentrations on the seven sensors of the electronic tongue (*n* = 3).

**Fig. 6:**
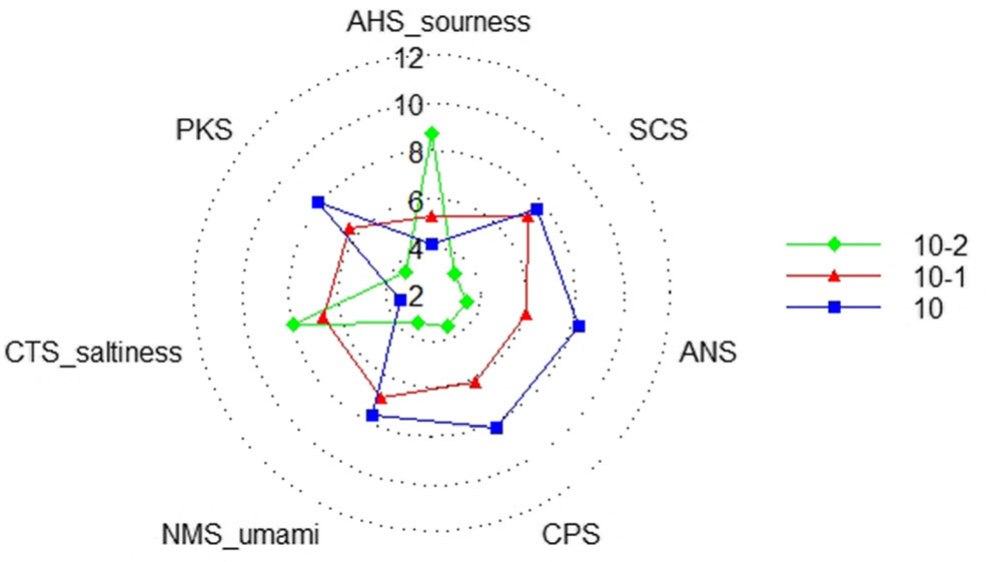
Radar map of relative taste intensity using the electronic tongue for different sample concentrations.

Figure 5 shows the mean values of the response signals based on the different concentrations of levetiracetam on the seven sensors of the electronic tongue. The signals of these samples could be distinguished on most sensors, especially on the AHS, CTS, PKS, ANS, and SCS. The deviation of the results after repeated experiments with three similar samples was minimal, indicating good reproducibility. Figure 6 shows the taste radar map of samples with different concentrations, and the values in the figure are the relative intensity of the taste of the samples on a scale of 0–12. The taste radar map helped observe the difference in taste between the samples, which indicated that the method can be a used as good indicator of the differences in taste of this product.

**Fig. 7:**
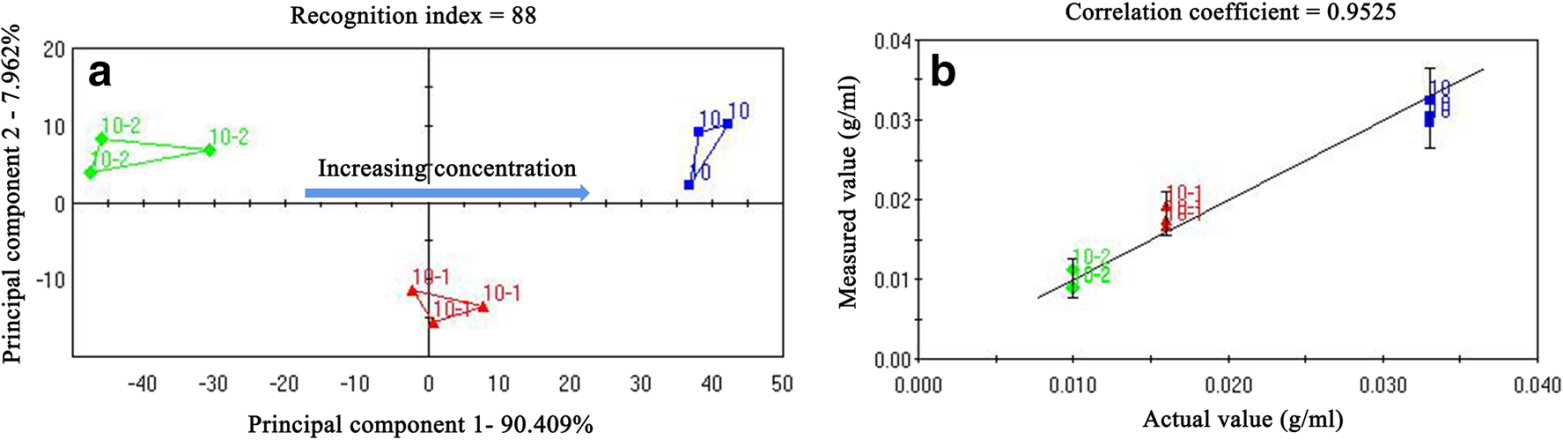
Plots of PCA and PLS regression analysis of the response signals on the electronic tongue by samples of different concentration (**a**): PCA; (**b**): PLS regression analysis).

#### Determination of Sample Concentration Using PCA and PLS Regression Analysis

Figure 7(a) shows the PCA of the response signals on the electronic tongue. The sum of the contributions of the principal component 1 (PC1) and principal component 2 (PC2) reached 98.371%, which reflected the true characteristics of the sample. The recognition index of the sample was 88, indicating that the three samples with different concentrations were well distinguished using PCA, and showed a regular distribution of PC1, with increasing concentration. The concentration curve based on PLS is shown in Fig. 7(b); the horizontal axis is the input concentration and the vertical axis is the fitted value of the instrument. The correlation coefficient of the concentration curve was 0.9525, indicating that levetiracetam had a good linear correlation within the concentration range of 10–33.3 mg/ml. It has also demonstrated that the electronic tongue can be well suited for the design and evaluation of taste-masking prescriptions of levetiracetam-based formulations.

The 3D printed IDTs are usually placed directly in the mouth and swallowed with a single sip of water (approximately 15 ml). Based on the results of the study, the concentration of the sample was determined to be 33.3 mg/ml, which is equivalent to dissolving a 500 mg tablet in 15 ml of water, similar to the fundamental method of the IDTs administration. This sample concentration provides the most realistic representation for the bitterness of the preparation.

### Analysis and Evaluation of Samples for the Designed Experiment

#### Sensor Response and Taste Analysis

**Fig. 8:**
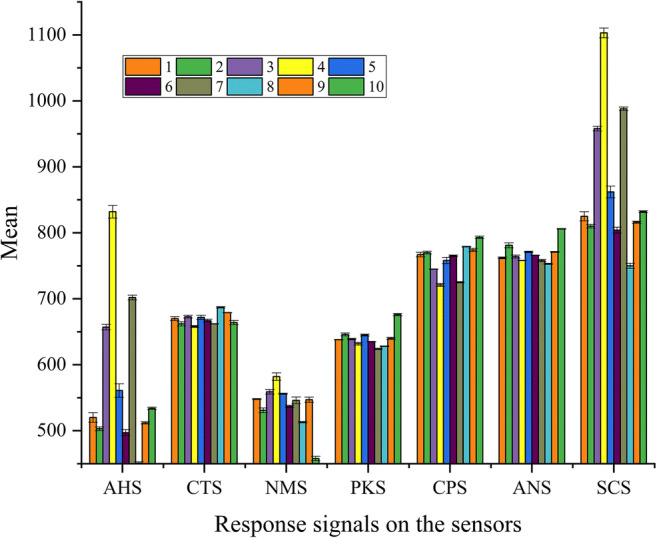
The response signals of sample no. 1–10 on the seven sensors of the electronic tongue (n = 3).

**Fig. 9:**
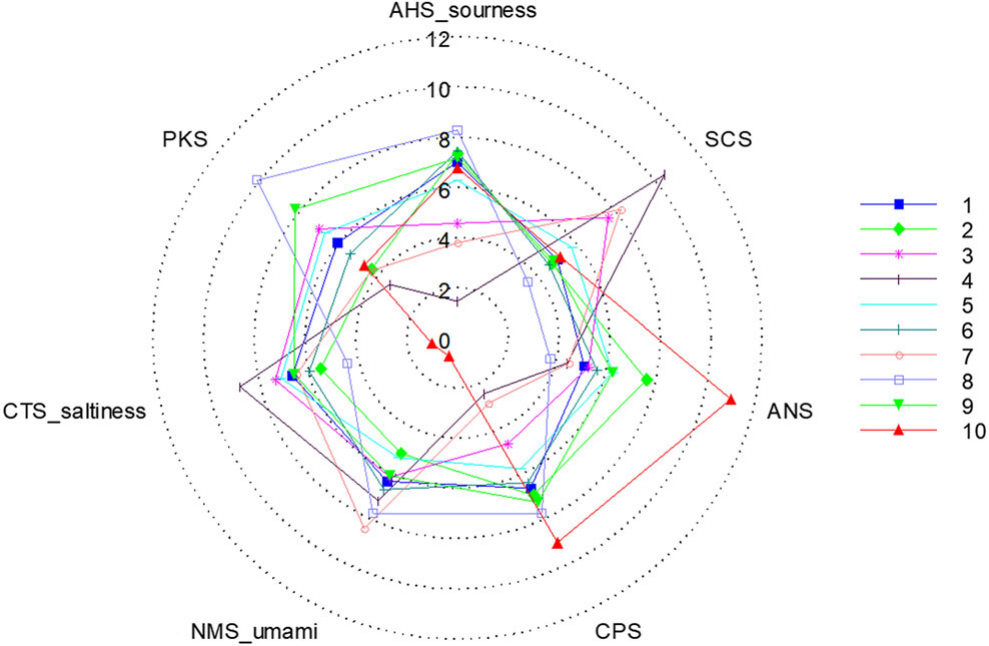
Radar map of relative taste intensity of the sample no. 1–10 using the electronic tongue.

Figures 8 and 9 show the mean values of the response signals of sample no. 1–10 on the sensors of the electronic tongue and the radar map of the relative intensity of taste, respectively. All the samples had a good response with slight deviation among the seven sensors, and the relative signal intensities of the different samples varied across all the taste sensors, especially on the AHS, CTS, CPS, ANS, and SCS. These signals allowed the ranking of the quantity of additives in different samples; although, considering the complexity of the sweet and bitter substances, more accurate conclusions were required in terms of PCA.

#### PCA of the Samples for the Designed Experiment

**Fig. 10:**
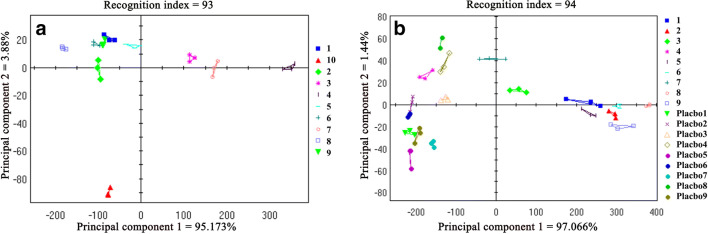
PCA to evaluate the relative distances between the samples for the designed experiment and the reference samples (**a**): PCA plot of the distances between sample no. 1–9 and the API; (**b**): PCA plot of the distances between sample no. 1–9 and the corresponding placebos).

**Fig. 11:**
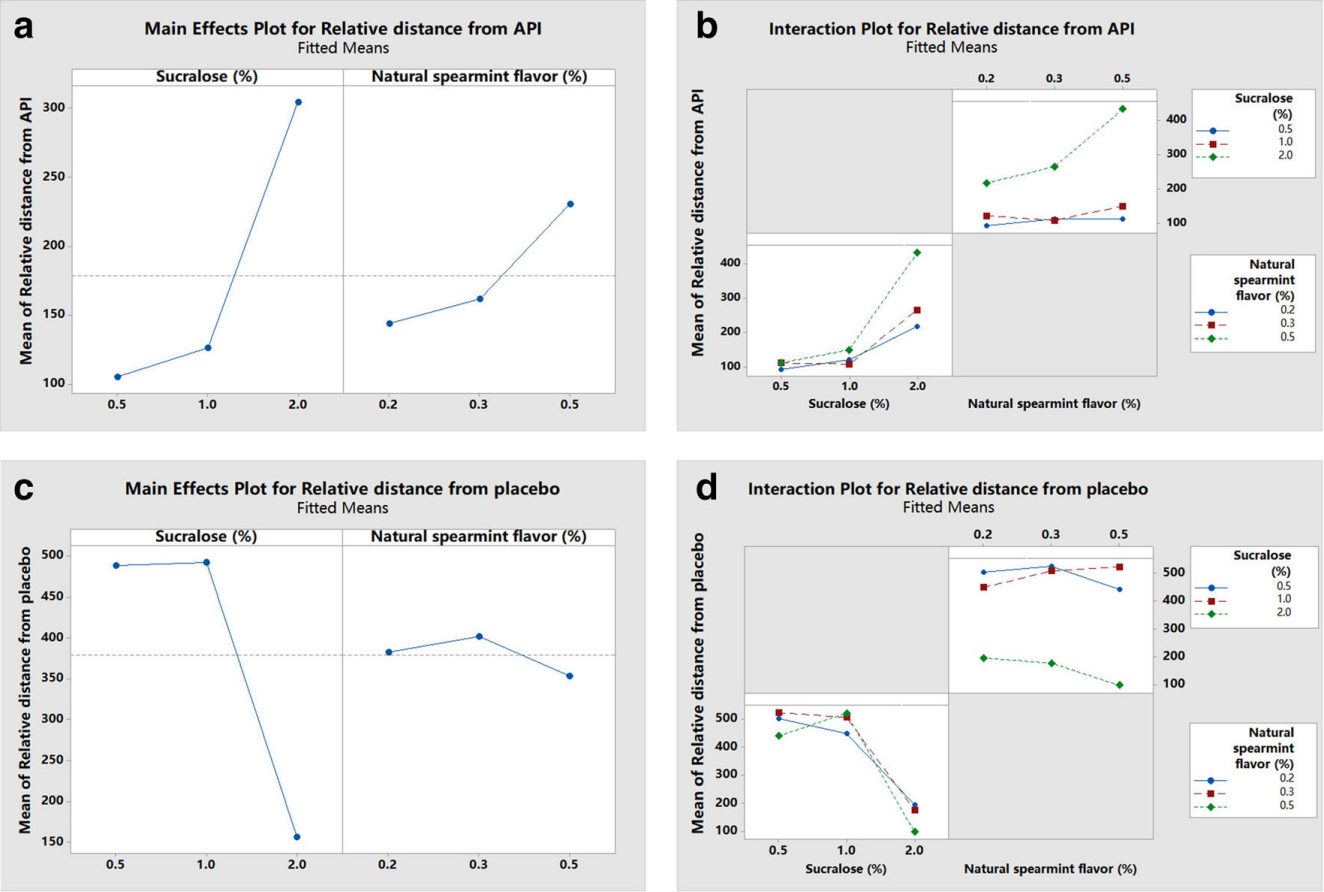
Main effects and interaction plots of the different quantities of the additives (**a**): main effects plot for relative distance from the API; (**b**): Interaction plot for relative distance from the API; (**c**): main effects plot for relative distance from placebo; (**d**): interaction plot for relative distance from placebo).

Figure 10(a) shows the PCA plot of the relative distances between the samples for the designed experiment and the API. The sum of the contributions of PC1 and PC2 reached 99.053%, and the recognition index of PCA was 93. This indicated that sample no. 1–9 were well distinguished from the API using the electronic tongue. Sample no. 4 was the furthest away from the API in the PCA plot, and had the greatest difference in taste. Table 3 (Y1) shows the relative distances between sample no. 1–9 and the API, and the relative distances were used as response values to analyze the results of the samples for the designed experiment Fig. 11(a, b). A main effects plot Fig. 11(a) shows that both sucralose and spearmint flavor had an interactive effect on taste, and the distance of taste from the API increases gradually with the increasing quantity of the two additives. The interaction plot Fig. 11(b) shows that when the quantity of sucralose was low (≤1%), the quantity of spearmint flavor (0.2–0.5%) had a decreased effect on the taste. But when the quantity of sucralose was high (2%), the effect of spearmint flavor on the taste was significantly enhanced; the greater the quantity of spearmint flavor, the farther the distance from the API, and better the taste. Similarly, as the quantity of spearmint flavor increased, the effect of sucralose on taste improvement also increased. Sucralose is one of the most desirable sweeteners at present because of its zero-calorie and high sweetness. It has a lightening effect on sour and salty tastes and a masking effect on astringent and bitter tastes. Sucralose can be used with spearmint flavor to confuse the brain's sense of taste and dilute the perception of bitterness through the nerve impulses generated by sweetness, aromatic odors, and bitterness aggregated in the central nervous system, thus providing a better taste masking effect. This study proved that the effects of the two additives on the palatability of the product were mutually reinforcing

### Model for Masking the Bitter Taste

This model was used to further determine the effect of the quantity of the additives on taste. Figure 10(b) shows the PCA plots of the samples for the designed experiment and the corresponding placebo. The sum of the contributions of PC1 and PC2 was 98.506, and the recognition index was 94; indicating that sample no. 1–9 as well as the corresponding placebo could be well distinguished. Sample no. 4 was closest to the corresponding placebo sample and had the best masking effect on bitterness. This result is the exact same as that mentioned in Section 3.2.2. Table III (Y2) shows the relative distances between sample no. 1–9 and the corresponding placebo, and the relative distances were used as response values to analyze the results of the samples for the designed experiment Fig. 11(c, d). The main effects plot Fig. 11(c) shows that sucralose had a greater effect on masking the bitterness than spearmint flavor, and the distance of the intensity of taste from the placebo decreased with increasing quantity of sucralose. The interaction plot Fig. 11(d) shows that when the quantity of sucralose was 0.5–1%, the taste of the sample was vastly different from that of the corresponding placebo and did not change significantly with the amount of spearmint flavor. When sucralose was 2%, the taste of the sample rapidly approached that of the corresponding placebo, and the distance was closest to the placebo when the quantity of spearmint flavor was 0.5%. Considering the results from the DoE and model for masking bitter taste, the best taste-masking effect was achieved with 2% sucralose and 0.5% spearmint flavor. The results of this study further demonstrate that the electronic tongue can be well used for the design of a taste-masked formulation of 3D-printed IDTs to improve palatability.

**Table III:** Results of the All-Factors Experimental Design

Sample no.	Y1: Relative distance from the API	Y2: Relative distance from placebo
1	110.95	440.36
2	93.16	503.71
3	216.37	195.60
4	432.25	97.30
5	121.13	448.30
6	111.49	523.66
7	264.90	176.51
8	149.82	522.32
9	108.33	507.21

### Taste Evaluation by the Human Gustatory Sensation Test

**Fig. 12:**
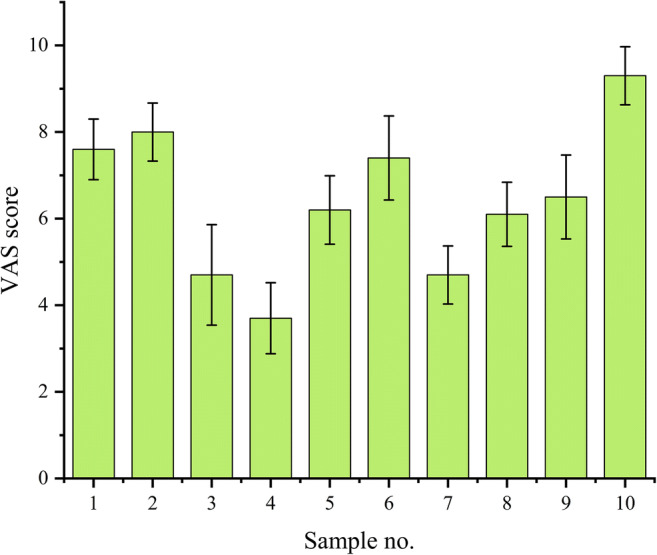
VAS scores obtained by the human gustatory sensation test.

In this study, the palatability of samples with different formulations was evaluated using the VAS. As shown in Fig. 12, a significant improvement in VAS scores with the addition of additives compared to non-taste masking formulation (sample no. 10) and sample no. 4 had the lowest score. The results indicated that sucralose had a greater effect on the improvement of palatability, with a significant improvement in taste as the quantity increased. The effect of spearmint flavor on taste was significantly enhanced when the quantity of sucralose was greater, and the greater the quantity of spearmint flavor, the better the taste. Overall, the results of human gustatory sensation test were consistent with the trend of the electronic tongue evaluation, indicating that the electronic tongue can be used for the taste evaluation of drugs to objectively assess and improve the palatability of the 3D-printed IDTs.

## Conclusion

In the development of new drugs and dosage forms, masking of bitterness is an effective way to improve drug compliance. In this study, BJ-3DP was used to successfully prepare IDTs that were dispersed within seconds and the IDTs were used for taste evaluation using the ASTREE electronic tongue. The results showed that the cross-inductance sensors of taste-sensing system could quantitatively evaluate the taste-masking effect on the 3D-printed IDTs. The taste-masking effects of sucralose and spearmint flavor were obtained using PCA analysis of the response signals in combination with DoE and model for masking bitter taste. The results proved that the effects of the two additives on the palatability of the IDTs were mutually reinforcing, and the optimal quantity of the additives was determined.

The flexibility of BJ-3DP allows for personalized formulations, such as different drug dosages and flavors, where taste evaluation via oral testing is impractical. The practical difficulties and ethical issues surrounding the development of pharmaceutical formulations limit the use of oral testing methods to evaluate the palatability of drugs. Our research shows that the electronic tongue can be used for the taste evaluation of drugs to objectively assess and improve the palatability of the 3D-printed IDTs, and contribute to the development of such 3D-printed formulations in the future. Due to the objectivity and reproducibility of the approach, the electronic tongue can be a promising tool for predicting taste perception and correcting deficits without the need for taste evaluation by a human panel. The combination of 3D printing and an electronic tongue helps to adjust the taste of pharmaceutical formulations to meet individual palatability requirements of a product.

### Acknowledgments and Disclosures

This work was supported by the National Natural Science Foundation of China (No. 82073793), the National Major Science and Technology Projects of China (No. 2018ZX09721003–007/ No. 2018ZX09J18107). The authors have no conflicts of interest to declare that are relevant to the content of this article.

## Abbreviations

API: Active pharmaceutical ingredient
BJ-3DP: Binder jet 3D printing
DoE: Design of experiments
IDT: Instant-dissolving tablets
MCC: Microcrystalline cellulose
PLS: Partial least square
PVP: Polyvinylpyrrolidone
PCA: Principal component analysis
VAS: Visual analogue scale
